# Supplementary material for: Characterization of Cellulose from Gagome Kelp and Its Effect on Dough, Gluten, and Starch as Novel Bread Improvers
Source: Foods. 2025 Apr 2;14(7):1246. doi: 10.3390/foods14071246 (PMC11988663; doi:10.3390/foods14071246)
Supplement: Supplementary file 1 [file foods-14-01246-s001.zip › foods-3505315-supplementary.pdf]

foods

MDPI

Supporting Information

Characterization of the cellulose from Gagome kelp and its effect on the dough, gluten and starch as novel bread improvers

Xiang Li [1,2,3], Heqi Yang [1,3], Xiaohui Yu [1]; Ying Tuo [1], Hui Zhou [1,2,3], Yidi Cai [1,2,3] and Long Wu [1,2,3,*]

[1] College of Food Science and Engineering, Dalian Ocean University, Dalian 116023, China
[2] Dalian Jinshiwan Laboratory, Dalian 116034, China
[3] National R&D Branch Center for Seaweed Processing, Dalian Ocean University, Dalian 116023, China
* Correspondence: wulong@dlou.edu.cn

Contents

Table S1 Sensory evaluated standard of the GC-bread SError! Bookmark not defined.

1. Table S2 Sensory-evaluation table of the GC-bread S4

Sensory Analysis Form

No. 2024102102 Participant number:

Table S1. Sensory evaluated standard of the GC-bread.

Items	Evaluation standard			
	**9～10 points**	**6～8 points**	**3～5 points**	**0～2 points**
**Color**	The color is uniform	The color is better	The color is dark and dull.	The color is dim and impurities
**Aroma**	The bread has baked aroma and no other odors.	The bread has light aroma, but no other odors.	The bread has insufficient smell with other odors.	The bread has sour smell with other odors.
**Tactility**	The bread shows well recovery after pressing.	The bread can recover after pressing.	The bread is hard to recover after pressing.	The bread is split after pressing.
**State**	The pores are uniform, and the structure is tight and uniform	The pores are uniform, and the structure is tight.	The pores are inhomogenous, and the structure is loose.	The pores are inhomogenous, the bread is loose and unformed.
**Shape**	The bread shows plump and complete shape	The bread shows a loose shape.	The bread shows a complete shape.	The bread shows an incomplete shape.
**Texture**	The bread is soft and punky, and shows no stick.	The bread is soft and slightly sticky.	The bread is hard and sticky.	The bread is very hard and sticky.
**Acceptability**	The bread tastes completely acceptable, and showing no obvious difference with commercial products.	The bread tastes almost acceptable, and same as commercial products.	The bread tastes hard to accept acceptability, and showing available difference with the commercial products.	The bread tastes non-acceptability, and shows significant difference with the commercial products.

Sensory Analysis Form

No. 2024102102 Participant number:

Table S2. Sensory-evaluation table of the GC-bread.

Items	Points				
	**No. 1**	**No.2**	**No. 3**	**No. 4**	**No. 5**
**Color**					
**Aroma**					
**Tactility**					
**State**					
**Shape**					
**Texture**					
**Acceptability**					
